# Cases in the Massachusetts General Hospital

**Published:** 1855-01

**Authors:** R. M. Hodges

**Affiliations:** Boston


### MEDICAL CASES AT THE MASSACHUSETTS GENERAL HOSPITAL.

REPORTED BY R. M. HODGES, M. D.

Case I.—*Puerperal Convulsions two months after delivery. Under the care of* Dr. M. S. Perry.—H. M. Irish. Married female. Entered Hospital, Aug. 2d, and was unable to give any account of herself. It was learned from a friend that she was confined two months since. The delivery was easy and natural, and her previous health had been good. She got up in about one week and put her hands into cold water to assist in washing; was taken with fever the next day and has kept her bed ever since, being delirious most of the time. Has had several attacks of convulsions lasting four or five minutes; but none for the last two weeks. Lochia persisted five weeks, gradually diminishing. Bowels constipated. Urine light colored.

Aug. 3d.—Nurse reports her to be sleepy, but easily roused, lying apparently insensible except when spoken to. Skin natural. Pulse feeble, 60. Pupils contract under the influence of light.—Respiration 24, and no unusual fullness of abdomen. Was ordered a cathartic and Hoffman's anodyne.

Aug. 4th.—After apparent improvement since previous visit, she had a severe convulsion lasting two or three minutes. She had had no dejection since entrance, nor passage of urine. She was therefore ordered an enema of soap suds and turpentine, and catheterism. R. Hydrarg. sub. mur., grs. ij.; ext. cicutæ, gr. j. M. Every four hours. If the convulsions returned she was to have ten leeches to temples. The head is to be kept cool and the feet warm.

5th.—Has had one convulsion. Now lies stupid and breathing stertoriously. Skin hot. Pulse 60, full. Pupils more dilated.—Cannot be roused. Swallows with difficulty. Having had no free dejection from the enema, is to have two dops of croton oil every three hours until free catharsis. Continue the pills and apply a blister to back of neck, and cataplasms to feet.

6th.—No convulsions; two free dejections after four doses of oil. Not quite so stupid as yesterday. Breathing less stertorous. Skin still hot. Pulse 84, soft and full. Pupils more contracted. Does not answer questions correctly. Micturition involuntary.—Continue pills and repeat the croton oil at night, if no dejection.

7th.—The patient was removed by friends, and died in two hours afterwards. No autopsy obtained.

The occurrence of convulsions after the termination of labor is usually looked upon as more dangerous than at any other period. This case was one of long duration, especially as there existed no congestion or other complication. Its cause must have been some injury to the nervous centres at the time of delivery, and which the erect position, fatigue and shock of cold water may have been instrumental in developing into an acute disease.

Case II.—*Gastro-Enteritis. Under the care of* Dr. M. S. Perry.—A. B., æt 47. Married. American, female. Entered Hospital Aug. 17th. Has kept a boarding-house, and worked hard for fourteen years past. General health good until one year ago, when she commenced suffering from languor, faintnesss, heartburn and oppression. For the last three months has been confined to room. Catamenia ceased, and coincidently a leucorrhœa of four years standing. Vomits upon taking the least nourishment, and says she has lost much flesh and strength. Complains of pulsation at epigastrium, which is tender, and where, to the left of median line, is discerned a tumor size of fist, which she reports to be sometimes on one side and sometimes on the other. She says it is the seat of cutting pains, and that these pains are accompanied by much flatulence. Bowels reguar. Sleeps poorly. Tongue not coated, but reddish. Pulse 92. Skin cool. She has no thoracic or cardiac disease. Countenance anæmic, sallow and much emaciated. She is ordered four drops of hydrocyanic acid, liquid farinaceous diet, and milk with brandy. At the next visit she reported no vomiting, but some diarrhœa of a watery character. The tumor had disappeared. On the 21st it was again present and noticed to be accompanied by pulsations, but her general condition was improved, hands warmer, and appetite such as to induce her to ask for bread and piece of beefsteak. Tinct. iodine to be applied to tumor daily.

Aug. 28th.—There has been no return of vomiting, but a watery diarrhœa has existed since its cessation. The tumor is readily found, but may always be dissipated by gentle pressure or friction.

Sept. 3d.—Opium having a very transient influence upon the diarrhœa, she was ordered sub. nitr. of bismuth, grs. x. once in six hours. This successfully arrested it in the course of a day or two, and was stopped because she complained of pain in the tumor which otherwise is as previously.

20th.—Vomiting returned after an indiscreet use of food.—Controlled as before by hydrocyanic acid. Tumor not at all apparent. Abdominal muscles remarkably tense.

24th.—Is put upon syr. fer. iod. From this time up to the date of her discharge, the general health improved; though occasional attacks of vomiting and diarrhœa occurred, they almost always were the sequence of indiscretions of diet. She continued her iron and tonics, such as porter, wine, &c., with manifest improvement to her anæmic condition, and went out Oct. 19th "relieved," without any tumor that could be felt.

The tumor at the epigastrium in this case was undoubtedly of an inflammatory character, the seat of which was the colon as indicated by its transient disappearance to the varying degree of flatulence, and its permanent disappearance to her improved general health. Though at first the symptoms which accompanied the tumor might have led to the supposition that it was malignant, the speedy removal of the vomiting and the general improvement of her health, together with the disappearance of the tumor, of course dissipated any such fears. Another point of interest is the arrest of the diarrhœa by the sub. nitr. of bismuth, a medicine which Dr. Perry has used with success in several cases of diarrhœa, especially in those assuming anything like a permanent character. It might have been stated in the history of this case that the patient consulted a "Nutritive physician," who, according to her statement, purged hor one hundred and forty times.

CASE III.—*Chronic Diarrhœa. Under the care of* Dr. D. H. STORER.—A. B. had had chronic diarrhœa for three years, having from twelve to twenty-four dejections in twenty-four hours, and at time of entrance twenty-four in twelve hours. At times these were accompanied by some blood. Treatment has been neglected, and such as he has followed been of no avail. He has some phthisical sypmtoms, but they are not active. He was ordered milk and lime water with crackers for diet, and a half grain of the oxide of silver once in four hours. Under this treatment his dejections came down to four stools in twenty-four hours. At the end of a week a half grain of opium was added to the silver, and now, at the end of a fortnight, the discharges are hardly more than of normal frequency. Between the 23d and 29th of August the discharges were reduced down to five and six per diem. About the 3d of September they were increased by a departure from his prescribed diet. But without change of treatment he went on improving till his dejections were reduced to two or three in the twenty-four hours. On the 15th he was discharged "much relieved."

CASE IV.—*Sub-acute Metritis. Under the care of* Dr. M. S. PERRY.—A. M. Married. Irish. Entered July 27th. Four months since confined with her first child, and has not been well since. A fortnight after confinement she got up and went about house; her milk ceased to flow shortly after, and febrile attacks followed. Catamenia have never returned. Has been confined to her bed since May, with the exception of last week, during which she has been about. Complains of pain in left hip and groin, extending to bowels and side, but this is less now than at first, when it was sharp and stabbing, accompanied by dysuria and sense of weighing and bearing down. Appetite not good, bowels regular. Tongue red and moist. Skin hot. Pulse 120. Abdomen full but soft, tender, and swollen in hypogastric region. No leucorrhœa or vaginal discharge.

July 29th.—A firm tumor above pubes was distinctly perceptible, extending upwards four inches in the median line, symmetrical in shape, and not particularly tender on pressure. Pain had been previously relieved by the following pills, viz: R. Ext. hyoscyami, grs. ij.; pil. hydrarg., grs. iij., and they are continued.

Aug. 2d.—After several days of seeming improvement, it is reported, "No dejection for three days, more tenderness at epigastrium. Swelling increased, quite tender on pressure. Has dysuria and less water than usual." On the 3d, the swelling was smaller and the dysuria less. Mercurial odor was noticed, and the pills of the 29th were omitted. 4th. Pain and dysuria again increased. Examination of urine revealed pus globules, epithelium, oxalate of lime in considerable quantities and albumen. Her condition continued varying in this way up to August 15th. Swelling and tenderness, dysuria and shooting pains alternating with comfort and ease, and from that date she was put upon a tonic course with the syr. ferri iod.

21st.—Improved. Vaginal examination reveals uterus enlarged and tender to touch. Abdominal tumor smaller. General condition better. Appetite good. Continue the iron.

Sept. 1st.—Tumor almost disappeared. Is sitting up and dressed, doing well. Sept. 10th.—She was discharged.

Case V.—On the 23d of August there entered a patient with dysuria and incontinence of urine of three months standing, who had been for some time under treatment, coming on after miscarriage and the sudden arrest of her catamenia. The os uteri was congested. There was vaginitis and cystitis. There was no ulceration of the os uteri. Immense relief followed the local treatment of the vaginitis. But nothing like a cure was effected until the patient was slightly mercurialized, the beneficial influence of which course of treatment was strikingly confirmed by its application to two other patients in the male ward, both of whom improved from the day that the specific effect of mercury showed itself, and were rapidly and permanently cured by its action.

A case of severe "lumbago" unaccompanied by marked uterine or vaginal disease, and with too marked a character to be supposed attributable to the slight leucorrhœa and vaginitis accompanying it, was nevertheless completely relieved by a few local applications of the nitrate of silver.—Another patient, afflicted with asthma appearing periodically, coincident with the catamenial epochs, was afforded a great degree of relief by the local treatment of a slight leucorrhœa and vaginal inflammation which were coexistent. These two cases, in connection with the previous one, afford excellent examples of the influence upon the general system of derangement in any of the offices of the genito-urinary apparatus, and the successful result which their detection may give to the treatment of obstinate complaints or disturbances of the general health obscure in their origin. In neither of the three cases was there ulceration of the os uteri.

A sponge bath of nitric acid water seems to have an excellent control over the night sweats of phthisical patients. In one instance from July 11th, the date of the first application, up to August 6th, there was entire immunity, when previously they had been of nightly occurrence. Subsequent repetition afforded similar relief.

The above observations were collected in the wards of Dr. M. S. Perry.

Boston, October 25th, 1854.

[*Boston Med. & Sur. Journal.*

